# A Treatise on the Diseases and Special Hygiene of Females

**Published:** 1845-07

**Authors:** 


Art. VII.—*A Treatise on the Diseases and Special Hygiène of Females.* By Colombat De L'Isére. Translated from the French, with additions. By Charles D. Meigs, M.D. Professor of Midwifery and the Diseases of Women and Children in Jefferson Medical College, Philadelphia; Member of the American Philosophical Society; of the Philadelphia College of Physicians; of the Philadelphia Medical Society; and Physician to the Lying-in Department of the Pennsylvania Hospital. *With wood cut Illustrations.*—Philadelphia: Lea & Blanchard. 1845. 8vo. pp. 703.

We devoted an article in our last number to the excellent work of Ashwell on the complaints of women. The volume before us is devoted to the same class of subjects, and is the production of one who, like the author of the former treatise, has seen much service in his profession, and reached an eminent position in it. One is the work of an Englishman, and the other the work of a Frenchman; both experienced, practical, learned men, who have written from the stores of observation made at the bed-side. The former work comes to us in the language used by the author; the latter appears under the disadvantages of a translation—and this may account for the obscurity in some of its descriptions of which we have heard complaints. But there is this further difference between the works, that Ashwell confines himself particularly to those complaints of females which are remedied by medicine, while Colombat has given to his treatise a surgical character, intending it to be a guide in those cases especially where an operation is demanded. While, therefore, they seem to cover the same ground they depart from each other so widely in numerous points, and afford instruction so diverse, but at the same time so important to the practitioner, that we shall run no risk of repeating what has been already said if we enter upon a somewhat extended notice of the treatise of M. Colombat De L'Isère.

The translator justly styles it a "very learned work." Every page bears testimony to the high attainments of the author. In this volume he has cited more than a thousand authorities. We say it not in admiration, nor as affording any proof that the work is especially fitted to assist the practitioner in the discharge of his arduous duties, but we state it as a fact to which both author and translator appear to have attached some importance, and which ought, therefore, to be made known. Besides, the circumstance is not without its interest to a certain class of readers, however it may fail to command the regard of the physician seeking only for practical knoweldge. The citation of authorities is of great convenience to the student or writer, who may wish to extend his researches upon any particular subject. And even the quotations from the Latin poets are to our taste; they are as flowers strewed along the rough pathway of science. We like the sententious sayings of Horace and Seneca, and are amused by the whimsical conjectures of Ovid and Lucretius. In a word, we have experienced in the perusal of Colombat something of that pleasure which one derives from reading Burton's Anatomy of Melancholy, the only book, according to the great lexicographer's own confession, that ever took Dr. Samuel Johnson out of bed two hours sooner than he wished to rise. This, we trust, with some will be a recommendation.

We will allow our author to explain for himself the plan of his work, which will afford a correct idea of its general scope. He says:

"To complete our prefatory sketch of the plan of this volume, let us remark, that previously to entering properly upon our task, we have given—1. Four chapters, comprising the history of the physical, moral and physiological changes that occur in women at different periods of life. 2. The varieties of conformation; the surgical anatomy of the sexual organs, and the sympathies of the womb. 3. The different methods of exploration of the genitalia, and the history of the speculum uteri. 4. The general causes and a synoptical table of the diseases of females. It should be likewise observed, that our anatomical and physiological details are confined to considerations applicable directly to pathology and therapeutics. Under the conviction that all epochs and all nations are tributary to medicine, and that to shut up this noble science within the narrow boundaries of an age or a nation, is to do it injustice, we have neglected none of the materials furnished by antiquity, by the middle ages, and by cotemporary medicine of all countries. It will be perceived, that in stating a variety of opinions, of experiments, and of curious cases, scattered throughout many volumes, numerous collections, and French and foreign journals, we have scrupulously quoted the sources whence they were obtained. Lastly, in order to render our work as complete as possible, we have brought it to a conclusion by a long chapter on the special hygiène of the female; and hoping thereby to secure the better attention of the reader, and especially to lessen the dryness of the descriptions, we have intercalated throughout the whole extent of the work, historical facts and many curious and interesting cases."

The description of the "phenomena of puberty," in the first chapter, is in the style of Buffon, and would be considered a little too highly colored to suit "the lovely dears" of this country, but we will not dispute its fidelity on that account; there may be, as Coleridge expressed it, such a thing as "*human* nature and *French* nature."

Under the head of the "phenomena of menstruation" our author refers to many curious cases and opinions, and among others to the popular one of lunar influence upon the catamenial revolution, which he quotes as reduced to a proverb by the poet:—

"Luna vetus vetulas, juvens nova luna repurgat."

We suppose that there is about as much foundation for this belief as for the other very general notion of the influence of that luminary upon the weather. Every day in the year, every hour, perhaps it might be assumed, the rains are descending upon some portions of the earth, while drought is prevailing in others; yet the moon is assumed to be the cause of both. And in like manner it brings on the "menstrual travail" in one woman, and postpones it in another. In one it induces the menses at the change, in another at the full, in some about the quarters, and in many during every day of the lunar month. It is a most potent luminary which can shed such influences over our earth and its inhabitants. And yet M. Colombat finds authorities enough in favor of this ancient opinion.

Of the recurrence of the catamenia after a long cessation, he gives the following example:

"The Duchess of D., celebrated as much for her wit as for her admirable literary talents, assured me that, having ceased to menstruate at 35, she supposed she had reached her *critical* age; the more especially as she became marriageable at an early age; but at about her 45th year, that is to say, after ten years of menstrual suppression, she again became regular. From that time the duchess, who is at present 53 years of age, has been as regular as she was in her youth."

Prof. Meigs subjoins a long note to the remarks of our author on the cause of the menstrual revolution. He adopts the theory of Negrier, Gendrin, and Lee, that it is a reproductive effort in the system of the human female, corresponding to the "*rut*" of the lower animals—that every such effort is attended by the rupture of a Graafian vesicle, and that this event constitutes the essential feature of menstruation, or, as Raciborski expresses it, "is menstruation itself." According to this view, "the menstrual hemorrhage is but a secondary phenomenon" in the function, the capital one consisting in the "maturation and the periodical discharge of the ova or the '*ponte*' (laying)." Prof. M. states the doctrine more at length in the following paragraph:

"But it is universally admitted that the human female, unlike the other mammals, has no stated season of reproductiveness; that she is liable to fecundation at any period of her reproductive age. Hence it appears clearly that a necessity exists for her evolving and perfecting the germina of her offspring, throughout the entire series of years from puberty until the arrival of her critical age. Supposing her to be perfectly regular, and never to become pregnant, she would, in the course of thirty years, have nearly four hundred menstruations. If, now, each menstruation is to be taken as the sign of her constitutional aptitude for fecundation, it is a just inference that such aptitude must bear a close relation to the perfectness of the germ-cell or ovulum of the Graafian vesicle. Let it be admitted, then, that a vesicle is always found to have been discharged in the cases of women dying suddenly in the menstrual act, and it does not seem to be a violent wresting of the facts to apply them to the solution of this great problem, the cause of menstruation. MM. Gendrin, Negrier, Lee, Raciborski, and Bischoff all concur in the statement that in menstruation the ovarian vesicle is burst, with loss of the ovula and granules or retinacula—that the ovarian stroma and indusium are highly injected and vascular—that a similar condition exists in the tube and womb, and thus is sufficiently established a hyperæmic status of the internal genitals, to account for the entire local plethora or uterine hyperæmia, whose result is the menstrual discharge. Here, at last, we see established the doctrine of a local plethora as a cause of menstruation, and that plethora resting on a sure physiological basis—the regular periodical development, to wit, of the Graafian vesicle with its contents."

The facts adduced in this passage account for the circumstance of which all practitioners must be apprised, that women sometimes become pregnant without having observed any show of the catamenia. Raciborski speaks of women who have given birth to several children without having ever seen the menses; and in such, he remarks, the menstrual act is confined to the expulsion of the germinal vesicle. The theory is an exceedingly interesting one, and we conceive that room is much more profitably occupied in giving an account of it than, after the fashion of M. Colombat, in citing the opinions of Hippocrates and Aristotle concerning the nature of the blood of the menses, or in quoting the ridiculous assertion of Pliny, "that it is a fatal poison, which deprives seeds of their fecundity, destroys insects, blasts the garden flowers and grapes, and causes fruits to fall from their branches." The periodicity of the discharge, which M. C. regards as so inexplicable, Prof. M. thinks, ought not to be deemed any more mysterious than the eruption of the first and second teeth, the growth and fall of the hair or the beard, or than "the periodical affections of the sphincters under a regularly operating law in physiology."

We have spoken of our author's powers of description; the following is the vivid picture which he draws of the "phenomena of gestation:"

"Scarcely has conception taken place when the vital forces of the womb assume new strength, and radiate to the rest of the economy the most extrordinary sympathies and the most powerful reaction. The relations of the womb with the brain appear to become more intimate; the circulatory, the digestive and the respiratory organs are subjected to an unusual stimulation, and their sympathy with the gestative organs, whether greater or less, is announced by syncope, convulsions, faintness, dyspnœa, disgusts, and perverted appetite; by spasmodic colic and a variety of other appearances which are also signs of pregnancy. The nervous susceptibility is augmented, the inclinations and passions are changed, the sensations become more acute, and the force of the intellectual faculties is sensibly increased on the one hand, or, on the other, lessened; the imagination is more excitable and the judgment less sound. Women have been found to become insane in pregnancy, some have become musicians or poets, and some have acquired a thievish propensity. Their will loses its strength; their affections are less constant; antipathies, anger and even cruelty are occasionally met with in the sex, whose natural and inherent inclinations are marked by gentleness, goodness, compassion, tenderness, an exquisite sensibility, and the most eager desire to console the unhappy."

In regard to the period of life at which the cessation of the menses occurs, Professor Meigs has given a tabular statement of 183 cases, which is as follows:

| | | | | | | | | |
|---|---|---|---|---|---|---|---|---|
| 21 | years | 2 | 37 | years | 4 | 48 | years | 8 |
| 24 | " | 1 | 38 | " | 7 | 49 | " | 7 |
| 26 | " | 1 | 39 | " | 1 | 50 | " | 12 |
| 27 | " | 1 | 40 | " | 18 | 51 | " | 4 |
| 28 | " | 1 | 41 | " | 10 | 52 | " | 8 |
| 29 | " | 1 | 42 | " | 7 | 53 | " | 2 |
| 31 | " | 3 | 43 | " | 4 | 54 | " | 5 |
| 32 | " | 2 | 44 | " | 13 | 55 | " | 2 |
| 34 | " | 4 | 45 | " | 13 | 56 | " | 2 |
| 35 | " | 6 | 46 | " | 9 | 57 | " | 2 |
| 36 | " | 7 | 47 | " | 13 | 60 | " | 1 |

M. Colombat, upon the authority of Orfila, relates the case of a woman who gave birth to seven children, the first at forty-seven, and the last at sixty, who was regular at her ninety-ninth year, and died at the advanced age of one hundred and fourteen. He cites other instances of fecundity in old age, but none quite so remarkable as this. Pliny, for example, knew a Roman matron who gave birth to a son at sixty-two, and the great Haller mentions a woman who was "still regular at seventy, and brought to bed at that age." The author himself had a relative, the mother of ten children, who never ceased to menstruate from the age of eighteen to her seventy-third year. Many females, his experience has taught him, are unwilling to confess that they have lost this characteristic function of their sex, and desire to dispute every inch of ground with the advance of old age:

"They invariably conceal the ravages of time, and converse with evident disquietude of the close of their spring-time of existence, even when the faded traces of beauty, and the stealing traces of grayness in the hair proclaim the approach of winter. Delivered from the discharge attached to the important functions of the uterus, the woman loses along with it the power of conceiving, and ceases thenceforth to exist as for the species."

This period safely over she is less exposed than man to the assault of many maladies, "and is more likely to run a long career of existence."

The second chapter of this learned work is devoted to "the varieties of conformation, the surgical anatomy of the genital organs, and the sympathies of the womb." Under the latter head, the author finds an opportunity of indulging his propensity to quote the ancients, and Plato's extravagant fancy is referred to, "that the womb is a wild beast that obeys no reason, but which, when its desires are unsated, wanders about within the body and excites all sorts of irregular motions." These sympathies are well known to every practitioner, as influencing all the diseases to which females are liable. M. Colombat refers to cases of aphonia produced by a sympathetic action of the womb. We have distinctly before our minds, at this time, a case of this kind which gave us much concern many years ago. We were at a loss what to make of it, and got our first conception of its pathology from Dr. George Thompson, of Tennessee, who had witnessed similar cases; he called it hysteria, a form of which it unquestionably was. The woman was of the middle age and hysterical, had borne six or seven children, and was then in the first months of pregnancy. The aphonia lasted during the period of utero-gestation, and was attended by a general loss of muscular power; but both voice and general strength returned shortly after the birth of her child.

Particular directions for "Touching," and for the use of the speculum are given in the third chapter, to which the translator has added some practical remarks on auscultation as a resource in cases of questionable pregnancy. He says—

"It is not to be doubted that the use of the stethoscope may, in some cases at least, enable us to detect the sensible signs of pregnancy, if the child be alive, as early as the end of the fourth month of gestation. By its use, also, we may be very correctly determined as to our course of action, since it reveals with clearness the state of the child's circulation. Now, if we find that the pulsations of the child in utero are becoming dangerously disordered, either by excessive precipitation of the heart's action, or extreme feebleness, irregularity or slowness of the same, it is manifest that we have possession of the means of deciding whether the security of the infant demands our intervention, in the way of some obstetrical operation, as the use of forceps, &c. So, also, where, in a bad labor, we have repeatedly recognized the situation and activity of the fœtal heart, if upon carefully seeking for them in the same place, it being impossible for them to have changed their place, we have the elements of an *opinion* as to some operation of cephalotomy, &c., which we might have been highly inclined to perform, but from our respect to the rights of the fœtus—rights which cease with the cessation of its life. Doubtless, also, by means of auscultation, we may gain great light as to the diagnosis of position, to the saving for the patient much of the distress or pain that an exploration with the whole hand could not fail to give; an exploration now often unnecessary, by the gentler intervention of auscultation.

"In the diagnosis of pregnancy from dropsy, and various other forms of disease, which, by their exterior physiognomy, so closely simulate several stages of gestation, the methods by auscultation are invaluable."

In the fourth chapter we have the "general causes and synoptical table of diseases of females." These the author divides into six sections: as 1. *Lesions of form and development.* 2. *Lesions of situation.* 3. *Physical lesions.* 4. *Vital lesions.* 5. *Lesions of functions.* 6. *Lesions appertaining to reproduction.*

Occlusion of the sexual organs constitutes a malady of the first class. It may be from union of the labia or of the nymphæ, from excessive development of the nymphæ, or of the clitoris, or from imperforation or other deformity of the vagina. A case of imperforate hymen is reported by Dr. Sutton on a preceding page of this number, in which the relief afforded by an operation was complete. But all cases of this description have not an equally favorable issue. Colombat states that the sudden escape of the fluid sometimes renders it impossible for the parietes of the womb, too suddenly emptied, to contract immediately, a circumstance that often gives rise to fatal inflammation, and to fever of a bad character. When very young in the study of our profession we remember to have heard our preceptor, the late Dr. Wilson Yandell, of Tennessee, relate the particulars of such a case. The subject, a young woman, had long suffered with retention of the menses before the nature of her complaint was suspected. When the operation, rendered necessary by the malformation, was performed, a dark colored, sanguineous fluid was freely discharged with the immediate relief of all the pressing symptoms. But the evacuation of the fluid was followed by great tenderness in the region of the womb, and other symptoms of metritis, from which the patient continued to suffer for many weeks. In the end, however, she recovered. To obviate the danger of a sudden evacuation the author proposes to make a small aperture in the hymen. The organs are thus permitted to return by degrees to their natural dimensions. But such a mode of procedure is not always admissible, and the membrane must then be freely incised. In some cases it is found to be thick, and extremely unyielding; in some, two membranes exist. The details of our author on all these points are curious, and will reward the careful perusal of the practical accoucheur.

In section second we come to our author's "lesions of situation," embracing all the displacements of the womb, various hernias of that organ, and of the vagina, vaginal cystocele and enterocele, vulvar enterocele and cystocele, descent of the internal membrane of the vagina, and invagination; in all, thirteen varieties. As in all other parts of his work the facts and authorities cited by M. Colombat on these heads are abundant, and of the former some are so startling that the translator deems it necessary to caution the reader against their too ready reception. Thus M. C., speaking of the possibility of conception in cases of prolapsus uteri, says—

"An incomplete descent of the womb does not prevent conception. We had charge of the case of an itinerant fishwoman, who become pregnant and was safely delivered, notwithstanding she had a prolapsus in which the tumor projected an inch beyond the labia. While she was lying down the womb retreated as much as eighteen or twenty lines within the vagina. Impregnation might possibly take place even in a case of almost complete procidentia, but in such a case the coitus must take place directly within the uterus itself."

Upon which Prof. M. remarks, "This is a statement that I look upon as wholly incredible."

M. Colombat proceeds:—

"Choppart cites, on the authority of Marignes, the instance of a female, who had, from the age of fourteen, been troubled with a prolapsus that slowly increased. The husband of this girl had no children by her until, after a considerable length of time, he dilated the orifice of the womb, and consummated the act of generation in its very cavity. The pregnancy was in all respects natural, but at the time of her confinement, they were compelled, on account of the rigidity of the os uteri, to make two incisions into it on opposite sides; after which the child was born dead, and the mother recovered without difficulty."

Prof. M. again interposes his caveat in the following terms:

"Such relations as the above require a stronger confirmation before they should be deemed credible. They are necessarily hypothetical as to the important steps of the doctrine."

"Pessaries and their varieties" are treated of in this connexion. The subject is a fruitful one, and has elicited no little contrariety of opinion, one practitioner declaring that "if he ever did any good in his life, it was by pessaries," while others of large experience and sound judgment discard them altogether. Professor Miller, of the Medical Institute of Louisville, after a long trial of the instrument in all its modifications, does not hesitate to express his doubts whether its use is ever beneficial or proper. Our author urges many objections to the use, or what the translator insists are the abuse, of the pessary; and Prof. Meigs admits that "great abuses are met with in the prescription and use of the instrument," but at the same time maintains, that "a great many persons are restored to health, and many preserve a tolerable state of health by their use, who, but for such aid would become irremediably diseased, or pass a long life of suffering. Beyond dispute, he adds, "there are women who can enjoy neither comfort nor health without the aid of these remedies, which, as our author states, are sanctioned by the common consent of the profession for ages past." We shall not attempt to decide in this controversy, but content ourselves with quoting from M. Colombat what may be said respecting the *choice of a pessary.*

"According to most authors, the round pessary is indicated where the diameter of the vagina is small; but, under contrary circumstances, choice is generally made of an oval, or gimblette, or figure of eight pessary.

"The stem or bilboquet pessary is chiefly used where the axes of the superior strait are very large, and the woman emaciated; or where the walls of the vagina, on account of their weakness and flaccidity, could not readily retain the instrument that is placed within them for supporting the womb.

"The pessary *à bondon* is reserved for cases in which the vagina is still prolapsed, notwithstanding the uterus itself is reduced. The elytroid pessaries, which, in many cases, supercede the others, are perhaps better suited than any others for the cases of anteversion and retroversion of the womb; in which we must raise that end highest that is designed to keep the womb in situ—of the particular deviations of which we shall hereafter treat. The cylindrical gum-elastic pessary of Dr. Rognetta fulfils the same indications perfectly well, and is especially excellent as a remedy for vaginal enterocele and cystocele.

"Where the patient has been troubled with a very copious uterine or vaginal catarrh, the use of the pessary ought to be deferred until this particular malady shall have been removed or at least much amended; for the presence within the vagina of such instruments could only increase the evil. In doing this we should but follow out the principle laid down by Boyer in his *Treatise on Surgical Diseases*, tom. x., where he says that the pessary should never be employed except in cases where the os uteri is neither engorged nor painful, and where we are certain that the symptoms experienced by the patient are dependent upon displacement of the womb, and not upon engorgement or elongation of its neck."

The "accidents connected with the presence of a pessary in the vagina" make a pretty long chapter, and are so formidable that they should "give" the young physician "pause" before deciding to introduce one. Thus, to sum up only a few, they never fail to excite irritation, they impede the functions of "defecation and urination," often produce insupportable pain in the loins and neighboring parts, without great cleanliness on the part of the woman they become incrusted with calcareous matter, "exciting inflammation and change of texture, ending in the formation of purulent vegetations that exhale the most repulsive odor," permit the neck of the womb to become engaged within the central opening, if that chance to be too large, and in this way give rise to strangulation "and the most serious consequences." More than all this:

"The action of the mucous apparatus being increased, acquires an exaggerated vitality that gives rise to vegetations that become so abundant as not only to fill the vagina, but to cover up the whole pessary and completely conceal it from the touch. Désormeaux, the father of the professor whose recent loss we deplore, was obliged to excise a great number of vegetations before he could succeed in extracting a pessary that had perforated both the bladder and the rectum. Professor J. Cloquet, in consultation in the case of a lady under treatment for cancer of the vagina, found the canal filled with fungous vegetations: having decided to remove these fungous masses, he discovered a pessary within the vagina and extracted it. The instrument which had been forgotten for 10 years, was completely covered with fungous matter and coated with a calcareous incrustation."

The complications contra-indicating the use of the pessary, and the indications of treatment in certain peculiar cases are pointed out, after which we have a discussion of the chances of cure. These are not great in the opinion of the author, but it is an interesting fact, as stated by the translator, that many women recover from very troblesome prolapsus, by a succeeding pregnancy.

Extirpation of the prolapsed womb, the *ultima ratio* of the surgeon in such cases, forms the subject of the next section. It is never to be resorted to except in the last extremity—*in evidenti mortis periculo*, but is then justifiable, as the experience both of ancient and modern surgeons abundantly testifies. A year or two since we published a case in this Journal in which the uterus was removed by Dr. Esselman, of Nashville, having been mistaken for a polypus, the result of which was fortunate. The method of performing the operation is given.

Respecting the cause of *anteversion and retroversion of the womb*, our author and his translator do not exactly coincide in their views. Both agree that while retroverson is an accident of almost daily occurrence, anteversion so rarely takes place that an accoucheur may pass through a long lifetime without meeting with more than a few cases of it. But they do not give the same reason for it. M. C. says—

"The explanation of the infrequency of the former and the frequency of the latter or retroverted state, is naturally found in the difference introduced by gestation into the relation of the parts. In fact, the posterior wall of the uterus, which, in the non-gravid state, is more convex than the anterior one, really dilates in pregnancy more rapidly than the anterior face, so that the fundus uteri naturally tends to follow the heaviest portion, which drags it downwards, that is to say, backwards, unless it is stopped by impinging on the face of the sacral curve. This is the reason why one of the principal predisposing causes is a too deep concavity of the sacrum. Another anatomical arrangement, which also tends to prevent the occurrence of anteversion in pregnancy is, that the anterior face of the womb, as it becomes more and more convex, encounters the symphysis pubis, and thus has a *point d'appui*, which tends to repel the organ in a backward direction. It is easy to understand the mechanism of retroversion, and the infrequency of anteverson during gestation, by reflecting that, on the one hand, the greater weight of the posterior wall of the womb draws the organ down towards the sacrum, and that, on the other hand, the retroversion takes place only because the cavity of the sacrum is excessive, allowing the womb to be jammed into it, either by a distended urinary bladder, or by the appui of the os uteri against the symphysis of the pubis; the broad ligaments, becoming shorter and shorter, should tend to hold up the body of the womb in the excavation, but the sacro-vertebral angle hinders its rise, and compels the fundus to incline backwards, and lodge at last in the hollow of the sacrum."

Prof. M.'s explanation of the phenomenon is as follows:

"I have met with a very considerable number of cases of retroversion of the womb, and though familiarly conversant with medical affairs for more than thirty years, I have not been able to meet with more than one single decided sample of anteversion of the organ. As I do not altogether agree with our author in the views he has presented us under this head, I shall take this opportunity to express, very briefly, my own opinions upon the subject.

"I should judge, from the great number of cases for which I have been consulted—cases coming to me from nearly every State in the Union—that great suffering is by many persons endured, under the idea that the patient has either a prolapsus, or an irritable uterus, or some derangement called disorder of the womb, and which is supposed to be curable by rest, or tonics, or sea-bathing; but which, in fact, can be cured only by the reposition of the dislocated organ. By inspecting the organs in *situ naturali*, on the anatomical subject, it may be clearly seen that the fundus uteri has a very free vibration backwards and forwards; and that it is only restrained from falling quite down, backwards, by the ligamenta rotunda, which, coming off from the angles of the uterus, and being inserted on the front of the pelvis, cannot permit a retroversion to take place, unless they are morbidly relaxed and extended. A woman who has a very large pelvis, and who allows her bladder to become enormously distended, will be always liable to retroversion during such distension, especially on the occurrence of any sudden effort or succussion of the abdominal muscles—as in a fit of sneezing, coughing, or laughter. A jump from a carriage-step, or a chair, or a trip on the pavement, while the womb is pushed backwards by the full bladder, may suddenly and even instantly jam the uterus under the promonotory of the sacrum, which, introducing a tenesmic feeling, is followed by bearing down efforts, every repetition of which aggravates the mischief. If the woman be non-gravid, perhaps she will empty the bladder, and the womb, raised upwards again by its anterior cords—its round ligaments—is not suspected to have been retroverted; but, if she be pregnant at two and a half or three and a half months, and the fundus be once jammed below the promonotory, it will probably remain there, even after the bladder shall have been perfectly emptied by the catheter. I have seen it remain so after the most complete evacuation of the urinary bladder, by the catheter. Let the reader think, for a moment, that, when the bladder of urine fills, it fills and distends backwards, not upwards; but it cannot contain a pint measure of urine without pushing the fundus backwards; and when the bladder can retreat no further in that direction, if the distension goes on, it rises upwards in the belly, towards the umbilicus, pushing the hypogaster outwards, whose curve is visibly augmented thereby.

"Let a woman two and a half months gone, get into a stage, or rail car, having neglected to empty the bladder beforehand; if she set off on her ride with eight or ten ounces in the organ, and is prevented for some hours from relieving herself, she will hardly reach her journey's end without having retroversion; and when she attempts to relieve the bladder, is found to labor under a total suppression of urine, or, at least a most painful dysury. I have seen such cases.

"A woman who has the habit of permitting large accumulations to take place in the bladder, can hardly fail in the long run, to relax and overstreatch her round ligaments so much as to render them at last useless to her. I am acquainted with more than one lady, whose round ligaments are so loose and useless, that the womb falls over into the hollow of the sacrum, from the slightest effort that she makes. I have had to reposit it many times, and, in doing so, have found the fundus turned quite down to the lower third of the sacrum. I do not think the weight of the superincumbent bowels has, in general, much, if anything, to do with producing retroversion. I look upon it rather as a case of relaxed round ligaments, and suppose that if there were any surgical means of shortening them, the womb, even one most prone to retroversion, would thereby be deprived of the liability to become retroverted; unfortunately we possess no such means. There can be little reason to doubt of the *contractility* of the round ligaments: certainly they seem, in some persons, to be at one time so relaxed as to allow the uterus to fall backwards with the greatest facility, and then they retain it for months in its natural situation; after which, they again permit the retroversion to take place again and again; to be succeeded by a period in which they are strong enough to prevent it, perhaps, during the remainder of the woman's lifetime.

"In retroversion, the os tincæ is drawn upwards behind and even above the top of the symphysis pubis. This state of the os uteri is attributable partly to the fact that the fundus, resting upon the lower portion of the sacrum, compels the other end of the organ to rise into the situation above mentioned; and this especially in such as have a gravid or otherwise enlarged womb. It should be remembered here that the pelvis, measured in an antero-posterior direction from the top of the symphysis to the lower third of the sacrum, is at least four and a half inches in length, but the womb itself is not more than three inches or three and a half inches in length. Hence, when the os uteri is forced up in the situation mentioned, it must be either because the womb is enlarged by pregnancy or by disease, or else because it is strained upwards in that direction by the contraction of its over-stretched round ligaments, which are now nearly parallel with the long diameter of the organ. Women, under retroversion, do certainly feel much pain in the groins and pubis from the strain on the ligamenta rotunda. I am very sure that a person in whom these ligaments are still in a healthful and natural state of tone will be extremely unlikely to have a retroverted womb; and that, where the accident has happened, it will only be necessary to give a slight help towards the reposition to make the ligaments draw the fundus upwards and forwards again, and retain it in *situ naturali* when once reposited."

For the treatment of retroversion we again quote from Prof. M.

"In certain cases of extreme difficulty encountered in the attempt to reduce the retroverted womb while the patient was lying upon the side or the back, I have readily effected the reposition, upon directing the woman to place herself upon her knees, with the thighs at right angles to the bed and perfectly vertical, while the top of the thorax, or rather sternum, should be in contact with the mattress. In such a position, not only is the weight of the viscera taken off, but what is of greater consequence, the power of tenesmic resistance is wholly abolished, while the position favors, in the highest degree, the reposition of the womb. In this posture the woman cannot bear down. I consider such a position as favoring the reduction in a degree far greater than the large bleedings recommended by Dr. Dewees, who bled ad deliquium for the purpose of abolishing the tenesmic power, or the power to bear down, which he considered as one of the chief obstacles to success. In such a position the surgeon can hardly fail of success, except in cases where reduction is rendered impossible from adhesions contracted in consequence of a long chronic state of retroversion—such cases are to be held as incurable."

We shall not attempt to follow our author through the details into which he enters concerning obliquities of the womb, nor to give an abstract of his views relative to inversion of that organ, to the consideration of which he devotes many pages. In like manner we shall pass without remark over the subjects, "elevation of the womb," "immobility of the womb," "hysterocele," "inguinal, crural, and ventral hysterocele," "hernia of the ovary," "vaginal cystocele, or hernia of the bladder in the vagina," "prolapsion of the mucous membrane of the urethra," "vaginal enterocele," and several other displacements, for the correction of which ample instructions are laid down under each head. And with the conclusion of these topics we reach the author's physical lesions.

To facilitate the study of the physical lesions of the external and internal genital organs of the female, he divides them into three classes:

"1. Contusions, wounds, and lacerations of the vulva, perineum, vagina, and uterus; ruptures of these organs; and finally contusions and wounds of the mammæ.

"2. Vesico-vaginal, utero-vaginal and recto-vaginal fistulas.

"3. The accidental introduction of foreign bodies into the genital cavities.

Rupture of the perineum is known to be a somewhat frequent, as it is a very distressing occurrence. Prof. Meigs entertains the belief that the proper support of the parts in labor would almost always prevent that accident, as will be seen by the subjoined extract:—

"The direction to support the perineum above given is insufficient; it should have been more explicit, as I am convinced that the instruction in the medical schools does not fully inform the young practitioner of the motive for the rule. The direction ought to be, to press the perineum in such a way as to make the head turn upwards in front of the symphysis in proportion as it emerges more and more from the pelvis. It should be to cause the head by supporting it, to move in coincidence with Professor Carus' circle. Now, Carus' circle, is a circle whose radius equals the semi-diameter of the pelvis, measured from front to rear. To draw this circle, take a pelvis and saw it through at the pubis and sacrum so as to divide them vertically. Set one leg of a compass on the posterior margin of the cut surface of the symphysis, open the other leg to half the diameter of the excavation, and with that leg describe a circle in the pelvis. This is Carus' circle, and it indicates the curved axis of the pelvis. It is along this axis that the centre of the head moves as it is born; and to hold the perineum, means to make it move until it is born, in coincidence with that line. When this is properly done, lacerations of the perineum rarely occur."

The author cites instances of delivery through an opening in the perineum, and the translator mentions a case in which, the head of the child being delivered, the attendant was unable to bring down the shoulders, and a surgeon was obliged to cut the anterior edge of the perineum before the labor could be accomplished.

When lacerations of the perineum remain uncured they can hardly fail to embitter the existence of the sufferer; yet examples are on record of women who, in spite of the calamity, have gone on to bear children and enjoy a decided degree of comfort. The following is one related by the translator, and we quote it, not as affording any apology to the surgeon for failing to direct all the resources of his art to the correction of the evil, but as holding out encouragement to those who may be the subjects of it.

"Many years ago, a lady here was delivered of her first child, with forceps, under the care of a distinguished practitioner, and suffered a frightful laceration of the perineum, from the effects of which she recovered her health after long and severe sufferings. She subsequently gave birth to two children, under the management of the same gentleman; after which I attended her in all her subsequent confinements, amounting to four.

"Knowing nothing of the peculiar nature of her situation, as she was in active labor, I Touched the child soon after arriving, and discovered nothing extraordinary. Sometime later, as the waters had gone off, I Touched again, and was shocked to find that there were fæces in the vagina, which, at first, I felt disposed to assign to a recto-vaginal rupture, which might have occurred, as I supposed, during some one of the violent throes that she was experiencing. The child was born, however; and, after the delivery of the placenta, I carefully examined the vagina. I now learned that what I had supposed to be a regularly formed vulva and vagina consisted of an opening extending from the posterior half of the anus to the anterior commissure of the vulva, or, what might very properly be called a cloaca; for the anterior perineum had wholly disappeared, and, along with it, all the anterior semi-circumference of the lower part of the rectum, whose anterior margin I felt up within the cloaca, and lying near the sacrum. Under such circumstances, of loss of substance and structure, had she now borne three children, and has had three others since that time. I am assured by her that, except when she labors under some diarrhœa or looseness of the bowels, she has not the least trouble on account of this new state of the parts; discharging her fæces at regular and stated times, and with the same facility as a person in health might enjoy. Moreover, her health is good."

Contusions, wounds and ruptures of the uterus, vaginal, vesico and recto-vaginal fistulas, with their appropriate mode of treatment, take up many pages. The author gives drawings of instruments, not as intelligible as they might be, by which he professes to be able to repair these fistulas with more certainty and safety than were attainable by the old methods of operating. The practitioner who may have the misfortune to be called upon in such a case will refer to the work for its specific directions. Under the head of "foreign bodies accidentally introduced into the vagina, the uterus, and the canal of the urethra," are recitals such as would not be met with in every work treating of the complaints of women, some of which exhibit poor human nature in a humiliating aspect. But if such things are done, some one must record them, and point out the remedy. The author quotes from Dupuytren, whose estimate of humanity was probably not a high one from all that was exhibited to his professional eye in the French metropolis.

We reach the "vital and organic lesions" of our author at about one-third way of his volume, but shall not attempt to give even the most superficial account of any but a few of them.

Prurigo of the vulva is an affection, by which most pregnant women have sometime during their lives been more or less seriously troubled. The author enumerates many remedies for it, but Prof. M. follows Dewees in preferring the borate of soda to every other. He says, he has rarely had occasion to order any thing more than the following formula, although a great many times consulted for the relief of this pruritus:

"℞ Soda borat. ℥ss.
Morphi sulphat. gr.vj.
Aq. rosæ distillat, ℥viij.
M. F. to sec. art. misturæ."

It is to be applied three times a day to the affected parts, by means of a sponge, or a piece of linen, the surfaces having previously been washed with tepid water and soap. The result of our experience is that the latter measure alone is sufficient in most cases of pruritus, if applied diligently and at an early period. We incline to believe that the intolerable itching is caused by a morbid secretion of the parts, for which soap and water are the best correctives.

After a few pages given to the consideration of the minor inflammatory affections to which the sexual organization of females disposes them, our author takes up *metritis*, or acute inflammation of the womb, which affection he separates from puerperal fever. With the translator, we deem this an injudicious arrangement, for these diseases, if not identical, resemble each other so closely, that the best view of them is obtained when they are placed in immediate connection. The subjects are of the first magnitude, and have received from M. Colombat and Prof. Meigs the consideration due to their importance. We shall be compelled to refer the reader to the work itself, since we could not profitably abridge these valuable chapters.

Cancer of the womb, prolapsus of the womb, cancer of the breast, and dropsy of the ovarium, are some of the disorders which occupy the author before he reaches the *lesions of the functions.* These embrace disorders of the menstruation, of which some account was given in our notice of Ashwell, hysteria, hysteralgia, and some other kindred affections.

In an elaborate note on hysteria Prof. M. indulges in certain speculations which are worthy of the examination of the physiologist. Perhaps the reader can comprehend these views from the following paragraphs:

"But it cannot be disputed, that the organs exercise each a special influence on the blood, that pabulum of the life and common bond of the members of it. The entire vascular system is lined with a membrane, called by Bichat, the *common membrane* of the sanguiferous system. But that membrane is by no means a unit. As the mucous membrane is not a unit, but is different, as it serves in different places to line organs wholly unlike in their nature and offices, so the common membrane of the blood vessels is everywhere different, as in the vessels of the brain, the liver, the stomach, the reproductive organs, &c.

"Let that membrane be regarded as constituting one single cavity or cyst, the angeiotenic cyst, containing, as in a simple single sac, all the blood of the subject, computed to amount to six hundred ounces. Let it, for the argument sake, be conceded, that the blood in this cyst, as is the fact, is perpetually changing its place of contact with the surface, so that the discs come to be placed in succession, in contact with the interior surface of every organ of the body; brain, lungs, stomach, womb, &c. Can we reasonably then doubt, that each one of these organs, so different in nature and power, taking out, from, and depositing in the sanguine mass the materials for its secretion, and the detritus of its substance, can we doubt, I say, of the powerful influence each one of them can exert on the constitution of the blood? Is it not true, that each organ can and does then modify the crasis of the blood, influence the hæmatosis, and thereby rule and domineer, when diseased, over the law of the life of the organisms, by this single power."

This conjecture, respecting the diverse nature of the sanguiferous membrane in the various vessels, has something plausible in it, but it rests upon too slender a basis of facts to give it any strong hold upon our confidence. Notwithstanding it may come to something; it is certainly ingenious.

The notes and comments of Prof. Meigs constitute a valuable portion of this work, and generally inculcate doctrines and practice to which the ablest American accoucheurs will subscribe. But one feature about them has struck us as objectionable, and that the sensible and learned author can easily correct. The obnoxious point to which we refer is a certain elation of style, which is out of place in a treatise designed to be put into the hands of students, and to be consulted by practioners in laborious business, who have not made the technicalities of their profession a matter of special study. The following passage contains examples of this fault, and perhaps in a higher degree than any other in the book, but the same fault mars the excellence of too many other passages, which it would be easy to cite. The meaning of the writer does not always shine out clearly through his language. In a word, his views, bordering occasionally upon the transcendental, are too often expressed in words not in common use, and not understood by the majority of readers. This must have been felt in reading some of the extracts already given, and is very palpable in that which is subjoined:

"The vast complicity of the reproductive nerves with the entire of the ganglionic system and the cerebro-spinal nerves therein displayed, would seem sufficient to remove all doubt as to the disturbing power, which, when modified by disease, that system could exert on the other members of the ganglionic innervation, as well as on the brain-nerves, and those of the spinal cord. It is scarcely to be deemed philosophical to look upon the working of the organisms, as a mere concurrence of a given catalogue of organs in carrying on the business of general life, by contributing each its part in the grand scheme of the hæmatosis, the calorification, the innervation, the assimilation, and secretion merely, and without reference to the constitutional influence that each member of the confederacy exerts on its co-members. It is like looking at the operations of a great commercial partnership, in which each partner does his part in the correspondence, the buying and selling merely, when, in fact, each such partner exerts a power to modify the activity of every other member of the concern, beyond and besides his special routine of daily toil. Now, in a community of vital organisms, the nature, whether physical or physiological of the substance called *liver*, is so special, so peculiar, that throughout the whole range of the tissues, there is no other substance at all like it—it has a peculiar power, a peculiar metabolic or changing power, which it exercises for the conservation of various other parts of the economy. The same holds good as to *brain*, spleen, the stomach, the kidneys, &c. Each one enjoys not an independent, but a special life, and therein possesses the power to modify and control, to a certain extent, all other special lives; and this, too, without relation to the mere secerning and circulating power it enjoys, but in a more extended degree, by virtue of that special and peculiar life, since doubtless the life even of every cytoblast is peculiar and special, and then *à fortiori* is it so as to the organic products of the cystoblasts that compose all our physical frame."

Of the philosophy of this passage we shall not stop to say anything, for this article must be brought to a close, but respecting its style we are persuaded there can be but one opinion. No one, we feel sure, will gainsay our judgment, that it ought to be put upon "a strongly alterative regimen;" and this the author may hesitate the less to do, because there is in his writings so much stamina that they will bear the system well. It is admitted that "a writer has no vocation to give his readers pain," and it is equally clear that he has no right to give them unnessary trouble. So correct a thinker, and so good a teacher as the author of this extract must exert a decided influence upon the medical mind of his country, and he ought to be careful, therefore, to set no example of evil tendency. If medical books generally were written after the fashion of the above passage, the study of medicine would be abandoned. We have admitted that it is the worst passage in the volume; we grant, further, that the subject is a difficult one, and that with what simplicity soever treated the reader would have trouble enough to follow the author through all his refinements. What then must be his case, when the inherent obscurity of the subject is aggravated by the most perplexed style? Clearly, it is hopeless. With this single exception, the labors of the editor upon this treatise are worthy of praise, and, the author himself doubtless would admit, have added substantially to its usefulness.

